# Disturbed Small-World Networks and Neurocognitive Function in Frontal Lobe Low-Grade Glioma Patients

**DOI:** 10.1371/journal.pone.0094095

**Published:** 2014-04-08

**Authors:** Qingling Huang, Rui Zhang, Xinhua Hu, Shangwen Ding, Jingguang Qian, Ting Lei, Xuan Cao, Ling Tao, Zhiyu Qian, Hongyi Liu

**Affiliations:** 1 Department of Radiology, Nanjing Brain Hospital Affiliated to Nanjing Medical University, Nanjing, China; 2 Department of Neurosurgery, Nanjing Brain Hospital Affiliated to Nanjing Medical University, Nanjing, China; 3 Department of Biomedical Engineering,Nanjing University of Aeronautics and Astronautics, Nanjing, China; 4 Department of Scientific Research, Nanjing Sport Institute, Nanjing, China; 5 Department of Statistic, University of Florida, Gainesville, Florida, United States of America; University of Maryland, College Park, United States of America

## Abstract

**Background:**

Brain tumor patients often associated with losses of the small-world configuration and neurocognitive functions before operations. However, few studies were performed on the impairments of frontal lobe low-grade gliomas (LGG) after tumor resection using small-world network features.

**Methodology/Principal Findings:**

To detect differences in the whole brain topology among LGG patients before and after operation, a combined study of neurocognitive assessment and graph theoretical network analysis of fMRI data was performed. We collected resting-state fMRI data of 12 carefully selected frontal lobe LGG patients before and after operation. We calculated the topological properties of brain functional networks in the 12 LGG, and compared with 12 healthy controls (HCs). We also applied Montreal Cognitive Assessment (MoCA) in a subset of patients (n = 12, including before and after operation groups) and HCs (n = 12). The resulting functional connectivity matrices were constructed for all 12 patients, and binary network analysis was performed. In the range of 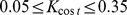, the functional networks in preoperative LGG and postoperative one both fitted the definition of small-worldness. We proposed 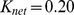 as small-world network interval, and the results showed that the topological properties were found to be disrupted in the two LGG groups, meanwhile the global efficiency increased and the local efficiency decreased. 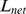 in the two LGG groups both were longer than HCs. 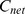 in the LGG groups were smaller than HCs. Compared with the Hcs, MoCA in the two LGG groups were lower than HCs with significant difference, and the disturbed networks in the LGG were negatively related to worse MoCA scores.

**Conclusions:**

Disturbed small-worldness preperty in the two LGG groups was found and widely spread in the strength and spatial organization of brain networks, and the alterated small-world network may be responsible for cognitive dysfunction in frontal lobe LGG patients.

## Introduction

Gliomas are primary brain tumors originated from glial tissue. As the disease progresses, most LGG patients are confronted with a loss of neurocognitive functioning which tends to have a global characteristic and cannot be explained by tumor localization alone [1], [2], [3]. Information interactions between interconnected brain regions are believed to be a basis of human cognitive processes [4], [5]. Higher neurocognitive functioning depends on both focal processing in different brain regions, and global integration of neuronal activity. Disturbances in resting state functional connectivity between remote brain areas have been demonstrated in patients with brain tumors. Brain activity of the different brain regions can be registered with the fMRI and electroencephalogram (EEG), which records electric fields related to intracellular neuronal currents showing dramatical changes [6]. The Wada test (intra-arterial amobarbital procedure, IAP) was used to determine language dominance and memory capacity in surgery candidates with temporal lobe epilepsy [7], [8], where the LGG group displayed significant differences compared with HCs regarding synchronization in different frequency bands, and associations of functional connectivity with neurocognitive functioning [9]. A study used the phase lag index (PLI) to evaluate changes in functional connectivity in brain tumor patients before and after operation under resting state magnetoencephalography (MEG) [10]. A significant decrease in theta band functional connectivity was found after surgery, which was hypothesized to be a result of a normalization due to the resection of the lesion. These results suggest that connectivity throughout the whole brain immediately reacts to changes in activity level in one part of the brain [4].

Recent studies based on resting fMRI have provided evidence supporting the neurocognitive disturbance of LGG in small-world networks [11], which is characterized by a high local interconnectedness (high C) and a short path length (low L) called a “small-world network” [12], [13]. Several studies have shown that both structural and functional brain networks in healthy humans and animals can be characterized by the small-world principle such as Alzheimer’s disease (AD) [14], schizophrenia [15], and brain tumors [16], [17]. Studies in brain tumor patients have shown that these patients display a loss of the small-world configuration and neurocognitive function, and functional connectivity has been associated with neurocognitive deficits in these patients before operation [16]. However, investigations regarding the impact of frontal lobe LGG after tumor resection on small-world network features in the human brain are relatively rare. In this study, we specifically focused on the frontal lobe LGG patients to directly investigate if these patients exist small-world topological property before and after operation under resting-state, and we expect to find evidence for an alteration of small-world network characteristics in pre–postoperative frontal lobes LGG which may be responsible for the altered neurocognitive function.

## Methods

### Subjects and Assessment

Between January 2011 and January 2012, only frontal lobe LGG patients which the gender, education, socio-economic status and age were matched with HCs in this study. Among them, 7 tumors located in the left hemisphere and 5 in the right side. The diagnosis criteria for LGG are:1)relatively small in size (the maximum size of LGG is 6.0 cm×5.0 cm×3.5 cm), 2)the extension of the tumor could not reach the central sulcus, 3) little edema in peritumor area, 4) slightly enhancement of tumor in CT or MRI with contrast, 5) excluding brain injury. All LGG patients were admitted for surgery in Nanjing Brain Hospital.They all had resting-state fMRI scan before operation and ten months after operation. HCs were recruited from the health staff of Nanjing Brain Hospital. All participants and HCs were right-handed. All subjects underwent a complete physical and neurological examination using an mild cognitive impairment battery of MoCA assessments. The neurocognitive tests were administered to each participant individually by a professional appraiser in the neuropsychological research center. The LGG was proved by the histological diagnosis. This study was approved by the Medical Research Ethics Committee of Nanjing Brain Hospital, and written consent was obtained from all participants. The fMRI and neurocognitive tests were recorded as part of regular patient care, and data were analyzed anonymously in this study.

### MRI Data Acquisition and Data Preprocessing

Images were scanned on Siemens verio 3.0 T superconducting MRI system in the department of radiology, Nanjing Brain Hospital. The structural scans were acquired using 3 D T1-Flair with the following parameters:repeat time (TR) = 1099 ms, echo time (TE) = 2.48 ms, time inversion (TI) = 900 ms, matrix = 246 x256, flip angle(FA) = 90°, thickness = 1 mm, gap = 0.5 mm, slices = 176.Resting-state BOLD-fMRI was collected axially using an echo-planar imaging (EPI) sequence with the following parameters: TR = 2000 ms, TE = 40 ms, FA = 80°, field of view (FOV) = 24 cm×24 cm, matrix = 64×64, NEX = 1, slices = 23, thickness = 4 mm, gap = 0.5 mm. The scan lasted for 500 seconds. The subjects were instructed to keep their eyes closed, relax their minds and remain as motionless as possible during the data acquisition. Rubber earplugs were used to reduce noise, and foam cushioning was used to fix the head to reduce motion artifacts.

**Figure 1:**
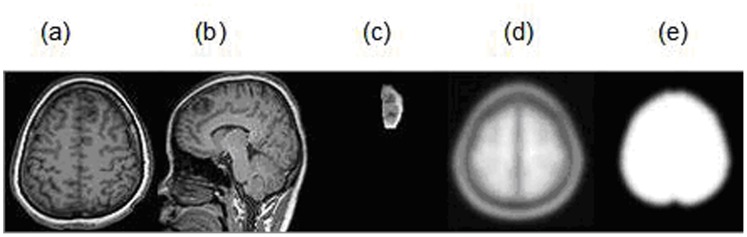
MRI images,mask and template images. (a),(b) axial and sagital structure images of a LGG patient in the left frontal lobe (T1 Flair), (c) masking image of left frontal LGG, (d) T1 template, (e) brainmask in SPM.

Data preprocessing was performed using the software package of SPM8 (http://www.fil.ion.ucl.ac.uk/spm).Slice-timing adjustment and realignment for head-motion correction were performed, and none of subjects was found to have excessive movement (translation exceeded 1.0 mm or rotation exceeded 1.0 u). We also evaluated the group differences in translation and rotation of head motion according to the formula of Liao [18].The results showed that the two groups had no significant differences (two sample t test, t = 1.512, P = 0.243 for translational motion and t = 1.821, P = 0.169 for rotational motion). We used cost function masking(CFM) to deal with the area of the lesion in spatial normalization.The functional images were then spatially normalized to stereotaxic coordinates of the standard Montreal Neurological Institute (MNI) and and resampled into voxel size of 3×3×3 mm^3^ (Figure 1), and then smoothed by convolution with an isotropic Gaussian kernel of 8 mm FWHW to decrease spatial noise. To further reduce the effects of confounding factors unlikely to be involved in specific regional correlation, we also removed several sources of spurious variance by linear regression, including six head motion parameters, and average signals from cerebrospinal fluid, white matter. Then, the residual time series were band filtered (0.01–0.08 Hz) to remove the effects of very-low-frequency drift and high frequency noise [15], [19].

### Anatomical Parcellation and Brain Network Construction

The registered fMRI data were segmented into 90 regions (45 for each hemisphere) using an automated anatomical labeling template, which has been used in several previous studies [15], [20], [21]. The Pearson correlation coefficients of each area were calculated for each pair of 90 functionally connected regions. Then we used a Fisher -to-z transformation to increase the normality of the correlation matrix, and got a binary connection matrix to make a graphic model of a brain network. The graphs were constructed over the whole range of connection densities or costs, from 5% to 35%, at 2.0% intervals. Global and nodal network properties were evaluated statistically over the range of 5–35%, and nodal properties were also analyzed at a connection density of 20%, among which the global efficiency showed the largest differences between the two LGG groups and the HCs. All other topological properties of the brain functional networks were calculated using software (Brat, www.ccm.org.cn/brat). These included the clustering coefficients (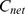), average path length (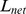), global efficiency (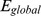) and local efficiency (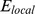), Lambda(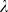), Gamma(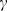), Sigma(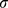), and these were evaluated statistically over the range of 5–35%, each of which has been described previously and used in several prior studies [15], [21].

### Statistical Analysis

All statistical analyses were performed using SPSS 15.0 for Windows (SPSS Inc., Chicago, IL, USA). The P value for gender distribution in the two groups was obtained by Fisher’s exact test. Independent-Samples t test was used for, age. MoCA scores The P values for education between the LGG groups and HCs were obtained by nonparametric test. MoCA scores between the two LGG groups was obtained by Paired-Samples t test. Statistical comparisons of 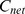, 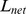, 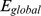, 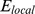), 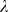, 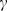, and 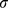 were performed separately, and significance was set at p<0.05. Correlation between the property parameters and MoCA scores was calculated by Bonferroni correction.

## Results

### Sociodemographic and Clinical Characteristics

There were no significant differences in gender, age, and education distribution between HCs and control groups (Table 1). However, there were significant differences of neuropsychological scores between two LGG patients and controls (preoperative LGG: p<0.001, t = 13.877; postoperative LGG: p<0.001, t = 12.369). Bonferroni correction for multiple comparions between MoCA and network characteristics were calculated respectively. p value of 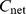, 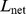, 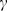, and 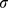 were all <0.05. Not one patient in the study underwent radiotherapy before the resting state fMRI scanning. None of the patients underwent radiotherapy after neurosurgery.

**Table 1:** Demographics and clinical data of LGG patients and healthy controls.

Characteristics		LGG patients (n = 12)		HCs(n = 12)	*t/z*	*p* value
		pre-LGG	post-LGG			
Sex (M/F)		5/7	5/7	6/6		>0.05^a^
Age (year)		43.2±13.1	43.2±13.1	41.8±12.9	0.251	>0.05^b^
education	primary school	4	4	3	0.488	>0.05^c^
	secondary school	5	5	5		
	high school	2	2	3		
	graduate	1	1	1		
MoCA		20.2±1.5	20.8±1.6	27.9±1.1	13.87712.369	<0.01^d^<0.01^e^
					2.548	<0.05^f^

a The P value for gender distribution in the two groups was obtained by Fisher’s exact test.

b The P values for age between the LGG groups and HCs were obtained by independent-Samples t test.

c The P values for education between the LGG groups and HCs were obtained by nonparametric test.

d The P values for MoCA scores between the pre-LGG groups and HCs were obtained by independent-Samples t test.

e The P values for MoCA scores between the post-LGG groups and HCs were obtained by independent-Samples t test.

f The P values for MoCA scores between the two LGG groups was obtained by Paired-Samples t test.

### Altered Topological Properties of Small-world Networks in LGG Subjects

**Figure 2:**
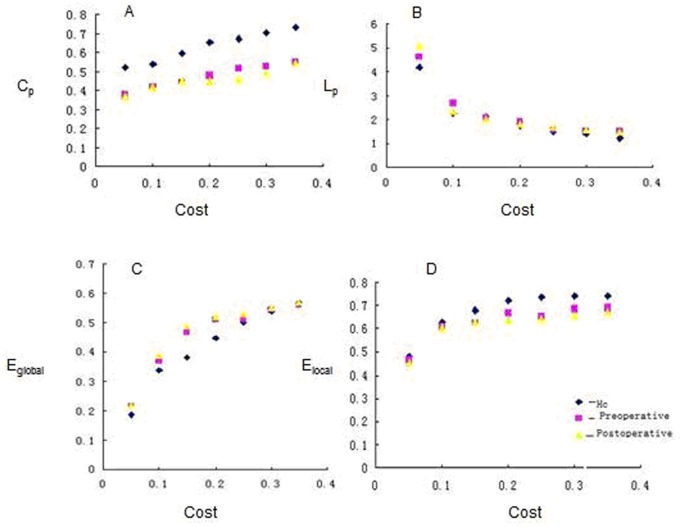
Change of network parameters as a function of connection density (Cost). Clustering coefficient (A), shortest path length (B), global efficiency (C) and local efficiency (D) of the HCs (blue point), preoperative LGG (pink point ) and postoperative groups(yellow point)as a function of Cost. The difference between the two LGG groups and the HCs, the preoperative LGG group and the postoperative are not significant at any Cost (P<0.05).

**Figure 3:**
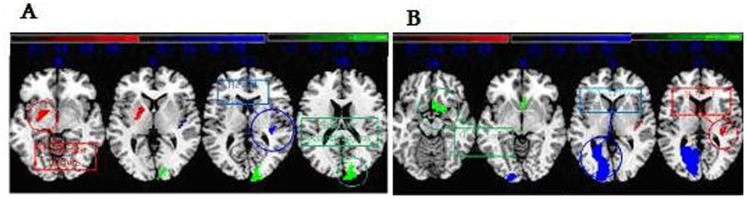
The maximum distribution of altered Cp at a connection density of 20%. Preoperative LGG (A), postoperative LGG (B).Colored bars indicate differences in network properties between the two LGG groups. Blue indicates regions showing an increase and red indicates regions showing a decrease in the two LGG groups.

The topological parameters were significantly different between LGG patients and HCs at the cost of 0.20(global efficiency showed the most prominent differences at this connection density). As shown in Figure 2A,B , the Lp of a network reflects how the network connects internally. In brain networks, the shortest path ensures the effective integration and fast transmission of information between distant brain areas. In the study, 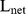 in preoperative LGG is shorter than the postoperative one, and the two LGG groups are all longer than HCs. 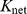 and 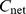 in the two LGG groups are both smaller than HCs. The altered 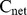 in the preoperative LGG includes the olfactory cortex, transverse temporal gyrus and clcarine sulcus, and the postoperative LGG display in lenticular nucleus, transverse temporal gyrus superior occipital gyrus (Figure 3).

**Figure 4:**
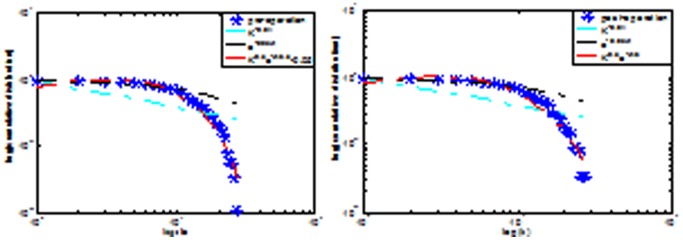
The cumulative probability distribution of nodes decreased in two lGG groups, and accompanied with increase of degree. Under the logarithmic coordinates cumulative probability distribution obeys functional form of 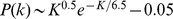 in the preoperative LGG (A), and that of 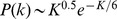 cumulative probability distributions in the postoperative LGG(B).

Nodes in the two LGG groups demonstrated significant differences. As shown in Figure 4, the regions with significant alterations are widely distributed across the brain, especially in the default mode network, which is composed of the cingulate gyrus, middle temporal gyrus, hippocampus and thalamus in the preoperative LGG, while in the postoperative LGG, the alterated different nodes includes the middle frontal gyrus, lenticula, paracentral lobule middle temporal gyrus which these areas revealed less connected functional brain networks in the study.

The average curve displays changes under the network cost (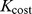) in global efficiency (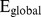) and local efficiency (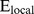) in the two LGG groups. As Cp increasing, 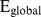 in the two LGG groups increased much more than HCs, while 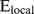 decreased in the two LGG groups than HCs (Figure 2 C,D). The overall efficiency in random network was greater than that of regular network, and the 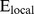 was less than that of regular network in the 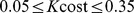 interval. The 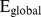 in the two LGG located between the random networks and the regular networks were consistent with property of small-world networks.

**Figure 5:**
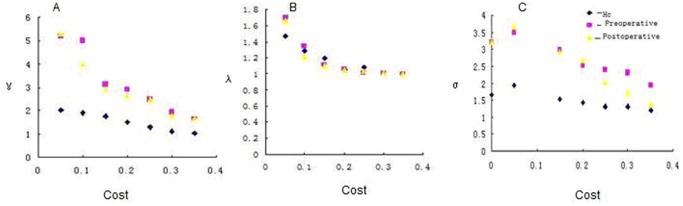
Change of small-world network definition parameters as a function of connection density. γ (A), λ (B), andσ (C) in the HCs (blue point), preoperative group (pink point) and postoperative (yellow point) as a function of cost. The difference between the LGG groups and the HCs is significant (P,<0.05).

The two LGG groups both fit γ = Cpreal/Cprand>1 and λ = Lpreal/Lprand≈1. Thus, the functional networks of the two LGG patients and HCs all fitted the definition of small-worldness [12]. Changes of network parameters were shown in Figure 5. 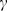, λ and σ values of the brain network played as a function of connection density.The values of 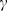 and σ were significantly higher in the two LGG groups than HCs. Meanwhile, the 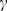 and σ values in the preoperative LGG group were larger than that of the postoperative LGG group, while 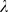 displayed few differences among the three groups.

### Correlation of Network Property and Neuropsychological Tests Scores

The significant differences of the network properties were observed, and a significant correlation was presented between the samll-world network property parameters and MoCA scores. Bonferroni correction analyses revealed that the alteration network property negatively correlated of 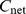,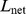, γ, λ, and σ. The MoCA scores showed positive correlation of 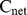 (P<0.05) (Fig. 2, 5). No correlations were presented between MoCA and network topology in the two LGG groups. These results indicate that disturbed networks in the LGG were related to worse MoCA scores (P>0.05) (Table 2).

**Table 2:** Smal-world networks property parametes and correlations between MoCA and network characteristics in LGG and HCs.

Characteristics	LGG patients (n = 12)				HCs(n = 12)	t	p value
	pre-LGG	post-LGG	t	p value			
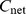	0.47±0.05	0.46±0.08	0.293	>0.05	0.63±0.10	4.953 4.675	<0.001<0.001
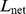	2.26±0.64	2.28±0.58	0.087	>0.05	1.64±0.65	2.354 2.544	0.028 0.018
γ	3.18±0.65	2.97±0.75	0.644	>0.05	1.52±0.72	5.872 4.823	<0.001<0.001
λ	1.17±0.33	1.16±0.23	0.070	>0.05	1.15±0.51	0.105 0.067	>0.05>0.05
σ	2.69±0.77	2.52±0.68	0.789	>0.05	1.48±0.48	4.617 4.318	<0.001<0.001

Bonferroni correction for multiple comparions between MoCA and network characteristicss.

## Discussion

Patients with cerebral lesions suffered from cognitive deficits, some of which could not be explained by local disturbance due to infiltration of the lesion [3]. Cognition requires a high level of functional interaction between brain regions to support daily activities. Cerebral lesions such as gliomas can lead to global alterations in functional interactions, even among brain regions remote from the tumor [4], [5]. The brain can be approached as a complex network of interacting brain regions [6], in which focal changes influence the integrity and functional status of the brain as a whole. Recent studies with noninvasive brain imaging technologies such as MRI, EEG, and MEG have demonstrated that the human brain’s structural and functional networks have small-world properties [22], [23]. The specific architecture of these small-world networks which combine local segregation with global integration, facilitate optimal (brain) network functioning [6], [9], [10], [11], and information processing in the brain depend on network interactions [15], [16]. In this study, we use a graph theory of small-world networks to compute the parameters such as the 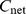, 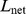, γ, λ,σ, and compare with the MoCA scores in frontal lobe LGG patients and HCs.

Under the threshold of 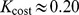, we compared 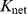,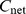,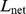,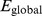,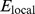 and property of the small-world parameters (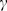, λ and σ) in order and random network in the three groups. We found that the preoperative LGG group and the postoperative LGG group both displayed a small world configuration, and the two groups did show changes in the overall organization of neural networks compared to HCs. This is especially interesting when comparing our results to previous studies in brain tumor patients, since sparse evidence suggests that brain networks become more random after the occurrence of lesions [22], [23]. The contradictory results in the present results may suggest that the human brain supports rapid and real-time integration of information across segregated brain regions after lesion resection. It maybe explains that the tumors included in the group were smaller in size, and with relatively benign pathological growth model, so it infiltrated less in depth and diamond. The network destroy were serious, then the two LGG groups show small-world networks property.

Shorter path length has also been demonstrated to promote effective interactions both between and across different cortical regions [24]. A longer path length may show more neurocognitive deficits, lower executive functioning and attentional task performance, also decreasing verbal memory in LGG [25]. Since path length remains shorter in this study, the two LGG groups both showed longer path length than HCs. Meanwhile, a higher score on the MoCA was correlated with shorter path length with poorer neurocognitive functioning. That the shorter path length in LGG was lower than the HCs indicated that the long distance information integration and transmission capacity of neurons was reduced in LGG patients [14], [22], [26]. Together with the lower global efficiency in LGG, these results suggest that information transfer between brain regions is more difficult in LGG patients. Our results are compatible with some previous contributions reporting predominantly in LGG group [26], [27], [28], [29].

Clustering coefficient and local efficiency of a graph system assess efficiency of communication between the first neighbors of a node when it is removed. 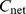 was the average of the clustering coefficients of all nodes. 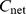 in the two LGG groups were smaller than HCs, as the same in lower global efficiency. The two LGG groups showed higher γ and σ of small-worldness properties than those of HCs. The most increasing regions of local clustering coefficient before operation were different from the postoperative one. The most distinguished regions in the preoperative LGG included the olfactory cortex, transverse temporal gyrus and the calcarine sulcus, while the regions in the postoperative LGG were lenticula, transverse temporal gyri and occipital gyrus. Brain networks with high clustering and high local efficiency are robust in local information processing even if some neurons are inefficient or damaged. Lower clustering coefficient and lower local efficiency imply relatively sparse local connectedness of brain functional networks. Higher network clustering coefficients indicate more concentrated clustering of local connections, stronger local information processing capacity [30], and associated with poorer verbal memory, and local clustering was significantly higher in LGG patients compared to HCs, which could be a compensatory mechanism. In our study, local clustering was significantly higher in LGG patients compared to HCs, which could be a compensatory mechanism. Some studies have reported inconsistent results regarding the topological properties of brain alterations in gliomas. A mixed group of glioma patients showed both lower clustering coefficients and shorter average path length than healthy controls pointing toward a more random network topology [17], but contradictory findings of MEG were reported in a more homogeneous group of LGG patients. These patients were of higher clustering compared with controls in the theta band, while the opposite was true in the beta band [16]. In a computational model study of functional effects of lesions [25], functional connectivity mainly decreased in the lesioned hemisphere, and significant changes herein were also reported in the contralateral hemisphere and the brain as a whole. Unfortunately, they have not investigated changes in overall network topology after structural lesions with small world network.


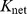
 is a measure to evaluate the degree of sparsity (or density, cost) of a network. Alterations in network architecture of degree were found in the two LGG groups. Node and degree of the two LGG group are not identical. Nodes in the cingulate gyrus, middle temporal gyrus, hippocampus and thalamus show less connectivity in the preoperative LGG, while in the postoperative LGG, nodes that were mainly located in the middle frontal gyrus, lenticula, paracentral lobule and the middle temporal gyrus exist less connectivity. A lower degree correlation was associated with decreased neurocognitive performance [23]. The phase lag index (PLI) was used to evaluate changes in functional connectivity in brain tumor patients before and after surgery [10]. A significant decrease in theta band functional connectivity was found after surgery, which was hypothesized to be a result of a normalization due to the resection of the lesion. It was suggested that these alterations may be associated with the global loss of neurocognitive function in patients. It can be expected that for optimal organization of a network, vertices with higher degrees are preferably interconnected. Preoperative maximal degree of brain functional areas included the posterior cingulate gyrus, the supramarginal gyrus, and insula, while in the postoperative LGG, precuneus, calcarine sulcus and superior frontal gyrus were as the same. Since the neurocognitive advanced features as a result of local and whole brain neurons function in the process of integration, the brain areas were associated with cognitive function, performing specific cognitive tasks. In our study, no difference was found in MoCA value between the postoperative LGG and preoperative LGG. Dynamic changes in modularity suggest that the underlying modular structure determines cognitive performance [28]. Several studies suggest that changes in connectivity and network topology may proceed during several phases, and plasticity is not stationary [31], [32]. Functional rehabilitation is strongly shaped by activity, revealing that a great variability of changes may occur at an individual level. Post-lesional recovery in monkeys is characterized by marked changes in both intra- and inter-areal changes in connectivity and network structure [33]. Differences in plasticity may occur between lesion types. It is highly probable that network changes induced by slow-growing tumors are fundamentally different from the effects of high grade glioma(HGG).

We have investigated the topological properties of human brain functional networks in homogeneous group of frontal lobe LGG using resting-state fMRI before and after operation. These results provide important evidence that brain network topology and cognitive decline also correlate with these network alterations. We confronted some troubles when processing the data. As the lesions presented, the images often mismatch between template and images on the site of the lesion, and the algorithms can lead to significant inappropriate image distortion and inaccurate functional location. So, we used cost function masking to reduce mismatch in spatial normalization of brain fMRI data with focal lesions, and improve the accuracy of brain functional location. The samples were relatively smaller, future studies with larger patient groups should elucidate in more details the interactions between these clinical characteristics, plasticity and network topology.
